# 肺癌引起Pourfour du petit综合征1例及其红外热像表现

**DOI:** 10.3779/j.issn.1009-3419.2012.01.14

**Published:** 2012-01-20

**Authors:** 大伟 张

**Affiliations:** 075000 张家口，中国人民解放军第二五一医院疼痛科 Department of Pain Clinic, PLA No.251 Hospital, Zhangjiakou 075000, China

## 1 病历摘要

患者，男，60岁。主诉左胸背痛伴左面部出汗增多4月余。患者于4个月前无明显诱因出现左侧胸背部疼痛，伴左侧面部出汗增多以及偶尔干咳。吸烟数十年，平均1包/日。查体：左肩胛骨内侧缘压痛（+），左锁骨中线第2、3肋间压痛（+），双肺呼吸音略粗糙，未闻及干湿性啰音。神经系统检查：左侧瞳孔直径4 mm，右侧瞳孔直径3 mm，双侧瞳孔对光反射灵敏，左侧睑裂稍大（上下睑缘分别位于角膜上下缘），右侧睑裂正常，左眼轻度前突（经眼球突出计测量，超过右眼2 mm），其余颅神经（-），四肢肌力Ⅴ级，肌张力适中，共济及感觉正常，双侧腱反射（++），病理反射（-），左面部出汗较右侧增多。64排CT肺部三维成像：左上肺野近肺尖部可见不规则软组织肿块影；右肺野清晰，双肺门未见改变，气管旁及主动脉窗下可见肿大淋巴结影（图 1）。红外热像：左侧额面部、左侧颈部及左上臂后面偏低温改变，考虑交感神经刺激征象（图 2）。入院后行超声引导下经皮肺穿刺活检术，病理检查结果为鳞状细胞癌，诊断为肺癌合并Pourfour du petit综合征。

**1:**
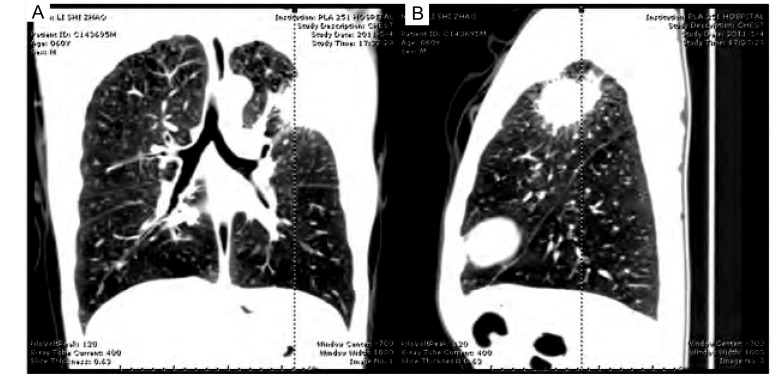
左上肺野近肺尖部可见不规则软组织肿块影；右肺野清晰，双肺门未见改变。 The left upper lung field near apex shows irregular soft tissue mass; The right lung is clear, bilateral hilus of lung shows no change.

**2:**
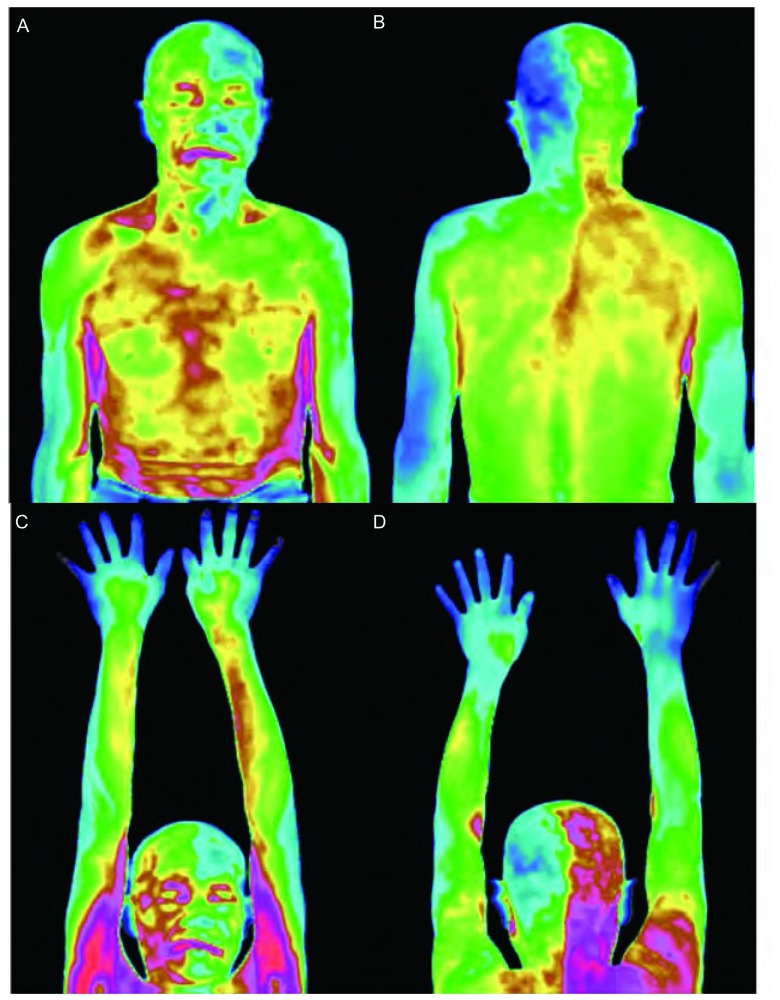
左侧额面部、左侧颈部及左上臂后面偏低温改变。 The left side of frontal-face, neck and the back of left upper arm show low temperature change.

## 2 讨论

Pourfour du petit综合征是以法国解剖学家、眼科医生Pourfour du petit（1664-1741）名字命名的颈交感神经综合征。系因交感神经传导径路中，自下丘脑交感神经中枢往下至支配头颈上肢段任一部位刺激性病损所致。其临床表现与Horner征（交感神经破坏性病损所致）恰恰相反，主要表现为病变侧瞳孔散大（瞳孔散大肌兴奋）、睑裂开大（睑板肌兴奋）、眼球突出（眼眶肌兴奋）、头颈上肢甚至半身皮肤血管收缩以及出汗增多^[1, 2]^。

引起Pourfour du petit综合征的肺部恶性肿瘤，多见于肺上沟瘤^[3]^。肺上沟（也叫锁骨下动脉沟）是锁骨下动脉对肺尖部形成的一处压迹，该部位附近的肿瘤称为肺上沟瘤（也可叫肺上沟癌）^[4]^。

肺上沟瘤多侵犯第7颈椎和第1胸椎外侧近脊柱部的交感神经节^[3]^，即星状神经节^[5]^。从解剖关系来看，胸膜顶位于第1胸椎椎体两侧，其内侧即为星状神经节^[6]^，所以肺尖部恶性肿瘤很容易侵犯星状神经节。在疾病早期，肿瘤刺激星状神经节，引起交感神经兴奋，出现Pourfour du petit综合征^[1]^。

该患者的临床表现较为典型，病变侧瞳孔散大、睑裂开大、眼球突出以及面部出汗增多。并且，通过红外热像检查，发现其左侧额面部、左侧颈部及左上臂后面均呈低温改变。提示这些低温区域是出汗增多和皮肤血管收缩的范围，因为此二者都可导致皮温降低。

红外热像技术是利用红外辐射照相原理研究体表温度分布状态的一种现代物理学检测技术，又称温差摄像。它能精确地记录出人体体表温度变化和分布形态，是研究人体温度变化、观察疾病的一项无创功能性检测技术。它对Pourfour du petit综合征的诊断价值在于能准确观察由皮肤血管收缩以及出汗增多所致皮温降低的区域。肺癌侵犯星状神经节恰好可以引起上述区域交感神经兴奋。

支配头颈上肢部位汗腺和血管的交感神经节前纤维来自脊髓T1-T4中间带外侧核^[7]^。其中支配上肢部位汗腺血管的交感神经节前纤维，在颈下神经节和上部胸节交换神经元，节后纤维沿臂丛、锁骨下动脉丛、腋动脉丛及上肢神经干走行；支配头颈部汗腺血管的交感神经节前纤维，在颈上、中、下神经节交换神经元，节后纤维经颈动脉丛和椎动脉丛走行^[5]^（图 3）。所以星状神经节受刺激后，同侧上肢部分区域（而非全部，因其节后神经元于星状神经节以下尚有一部分胸节）出现偏低温改变，系皮肤血管收缩及汗液分泌增多所致。而支配头颈部汗腺血管的交感神经纤维，因全部经过星状神经节，故出现病变侧偏低温改变。

**3:**
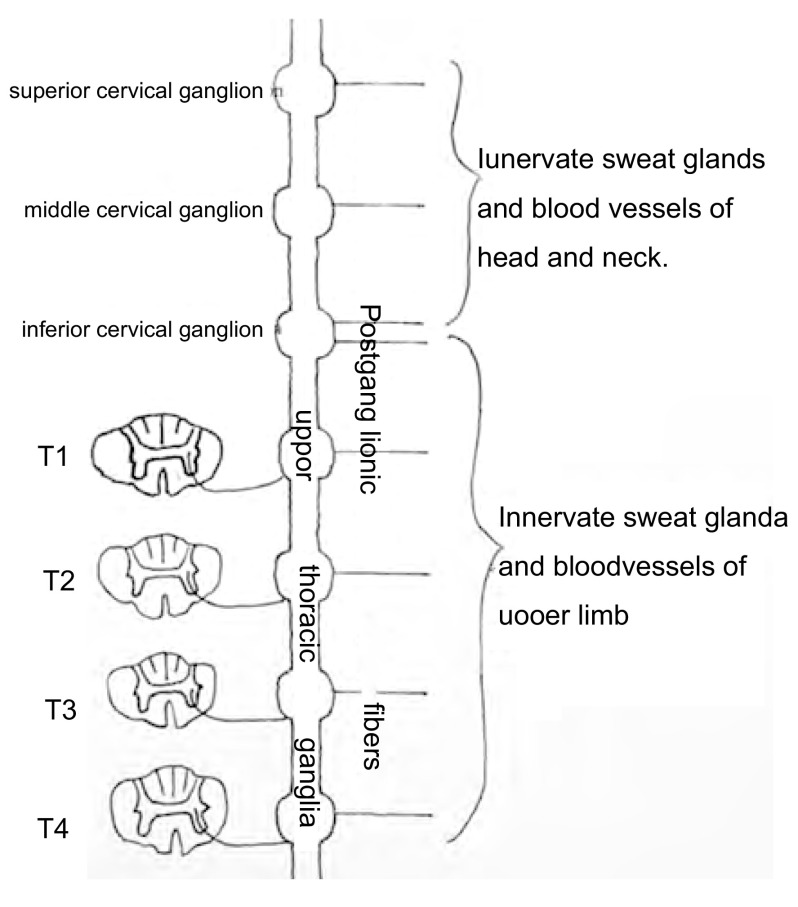
支配头颈部及上肢汗腺、血管的交感神经纤维走形示意图 The schematic diagram of sympathetic fibers that innervate head-neck and upper limb's sweat glands and blood vessels
